# Macrocyclic Compounds for Drug and Gene Delivery in Immune-Modulating Therapy

**DOI:** 10.3390/ijms20092097

**Published:** 2019-04-28

**Authors:** Hongzhen Bai, Jianwei Wang, Zhongbao Li, Guping Tang

**Affiliations:** Department of Chemistry, Zhejiang University, Hangzhou 310028, China; wjw126hz@zju.edu.cn (J.W.); 21637025@zju.edu.cn (Z.L.)

**Keywords:** macrocycliccompounds, drug and gene delivery, physicochemical properties, immunity mechanisms, immune-modulating therapy

## Abstract

For decades, macrocyclic compounds have been widely applied in various fields owing to essential physicochemical properties such as their rigid cyclic structures, geometric dimensions (diameter and height), hydrophobic cavity, and hydrophilic interface. This review is an attempt to summarize various research accomplishments involving macrocyclic compounds for drug and gene delivery in immune-modulating therapies: the structures and benefits of main host molecules, their mechanisms regulating the immune system from cell uptake to activation of dendritic cells and T helper lymphocytes, as well as their potential immunotherapy for different diseases. Macrocyclic compounds including cucurbiturils (CBs), calixarenes, pillararenes, cyclodextrins (CyDs), macrocyclic peptides and metallo-supramolecular compounds, have their own unique physicochemical properties and functional derivatizations that enable to improve the biocompatibility, responsiveness to stimuli, and effectiveness of immune-modulating therapy. Based on abundant clarifications of the biological immunity mechanisms, representative constructions of macrocyclic compounds for immune therapies have been conducted for the investigation of treatment of different diseases including cancer, atherosclerosis, Niemann-Pick type C1 disease (NPC1), diabetes, and inflammations. Although there are critical challenges that remain to be conquered, we believe the future of macrocyclic compounds in the immune-modulating therapy must be bright.

## 1. Introduction

**Figure 1:**
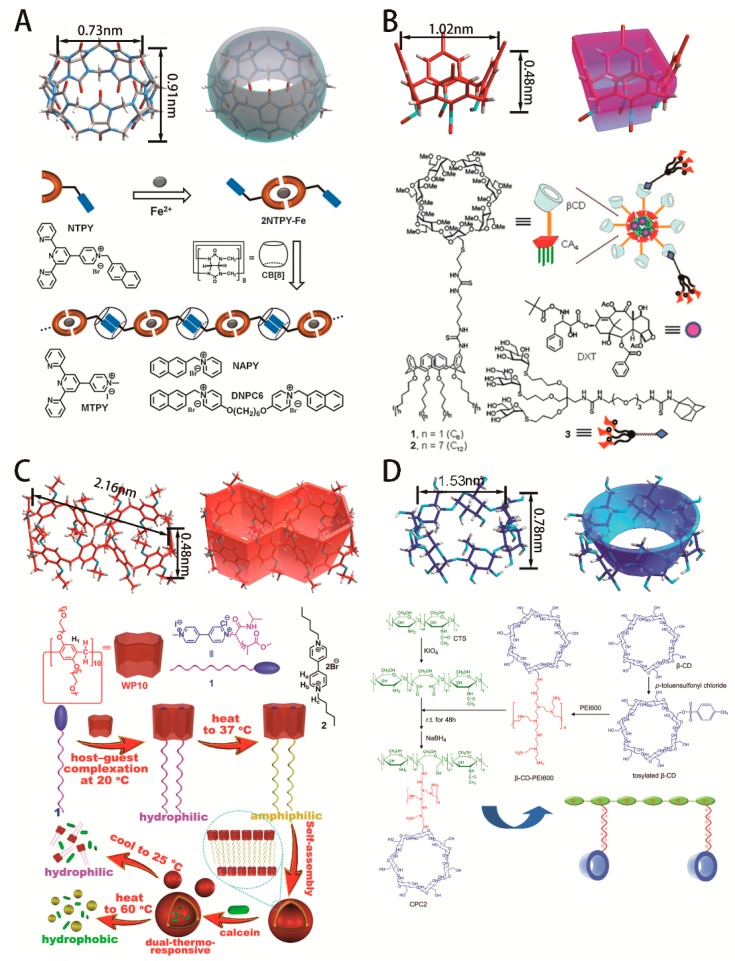
Macrocyclic compounds’ structures and applications: (**A**) Cucurbituril: Terpyridine-metal coordination to promote supramolecular polymerization and solubility employed by Zhang et al. (republished with permission of Royal Society of Chemistry, from Cucurbit[8]uril-based supramolecular polymers: promoting supramolecular polymerizationby metal-coordination, Zhang et al., 49, 5766–5768, 2013) [16]. (**B**) Calixarene: Glycoligand-targeted core–shell nanospheres with tunable drug release profiles from calixarene–cyclodextrin heterodimers developed by Fernández et al. (republished with permission of Royal Society of Chemistry, from Glycoligand-targeted core–shell nanospheres with tunable drug release profiles from calixarene–cyclodextrin heterodimers, Garcı´a Fernández et al., 50, 7440–7443, 2014) [17]. (**C**) Pillararene: A thermoresponsive supra-amphiphilic complexation based on Pillar[10]arene/Paraquat cooperative complexation designed by Huang et al. (Reprint with permission from J. Am. Chem. Soc. 2016, 138, 3168–3174. Copyright © 2016, American Chemical Society) [18]. (**D**) Cyclodextrin: Chitosan-graft-(polyethylenimine-β-cyclodextrin) cationic copolymers with high gene transfection and silencing efficiency synthesized by Li et al. (Reprinted from Biomaterials, 32, Li et al., Chitosan-graft-(PEI-β-cyclodextrin) copolymers and their supramolecular PEGylation for DNA and siRNA delivery, 8328–8341, 2011, with permission from Elsevier) [19].

In recent decades, controlled drug delivery systems have received extensive attention as they show great potential to solve lots of issues associated with conventional therapeutic agents and manifest excellent therapeutic effects in treating kinds of cancers and other diseases [1,2,3,4]. Among these platforms for controlled drug delivery [5,6,7,8,9], macrocyclic compounds have attracted increasing interest with remarkable developments in nanomedicine. Their physicochemical properties endow macrocyclic compounds with unique capabilities for nucleic acids and drugs delivery in a wide range of fields (illustrated in Figure 1) [10]. The geometric dimensions (diameter and height) of macrocyclic cavities require guest moieties of a suitable molecular size [11]. Other significant aspects include the hydrophobic cavity and hydrophilic interface: the former is responsible for drug entrapment via hydrophobic and van der Waals interactions, while the latter contributes to functional derivatization that enables responsiveness to stimuli such as pH, Reactive Oxygen Species ROS) and redox microenvironment [12]. Additionally, rigid macrocyclic structures have advantages over linear structures in certain essential properties, such as membrane permeability, metabolic stability, and overall pharmacokinetics [13,14,15]. All these structural properties and various functions improve the stability, biocompatibility, the drug-loading capacity and tissular permeation of the drug delivery systems, as well as the effectiveness and safety in immune-modulating therapy.

## 2. Macrocyclic Compounds

**Figure 2:**
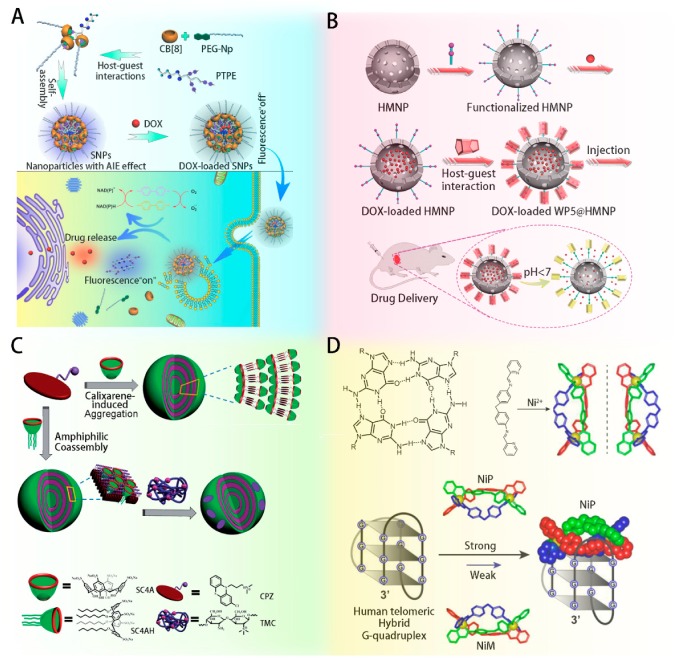
(**A**) The preparation of nanoparticles based on cucurbiturils and schematic illustration of the imaging-guided drug delivery reported by Chen et al. (Reprint with permission from ACS Appl. Mater. Interfaces 2017, 9, 44392–44401. Copyright © 2017, American Chemical Society) [20]. (**B**) Fabrication of pH-responsive mechanized hollow mesoporous nanoparticles (HMPS) based on water-soluble pillar[5]arene (WP5) for drug delivery in vitro and in vivo reported by Huang et al. (republished with permission of Royal Society of Chemistry, from Improved in vivo tumor therapy via host–guest complexation, Huang et al., 4, 2691–2696, 2016) [21]. (**C**) Principle of coassembly of an amphiphilic drug with calixarenes reported by Liu et al. (republished with permission of Royal Society of Chemistry, from Supra-amphiphilic aggregates formed by p-sulfonatocalix[4]arenes and the antipsychotic drug chlorpromazine, Liu et al., 10, 2253–2263, 2014) [22]. (**D**) The structures of NiP/NiM and representative illustration of NiP selectively recognizing of human telomere G4 DNA reported by Qu et al. (Reprint with permission from J. Am. Chem. Soc. 2017, 139, 16201–16209. Copyright © 2017, American Chemical Society) [23].

The main host molecules reported in the literature include cucurbiturils (CBs), calixarenes and pillararenes, cyclodextrins (CyDs), and several new structures such as macrocyclic peptides and metallo-supramolecular compounds (shown in Figure 2) [16,17,18,19].

CBs, unique pumpkin-shaped macrocyclic compounds bearing five or more glycoluril units linked by methylene bridges, possess hydrophobic interior cavities and polar carbonyl groups surrounding two identical portals [24]. CB-based host-guest interactions possess high binding constants owing to abundant hydrogen bonding, but cucurbit[8]uril (CB[8])-based supramolecular polymers with high molecular weight have poor solubility. Many excellent works have sought to overcome the major obstacles and develop various applications including cancer therapy and protein recognition in immunotherapy. For example, Chen et al. constructed an amphiphilic brush copolymer from CB[8] for cancer therapy (shown in Figure 2A) [20]. CB[8] has been shown to improve the stability of the building blocks in aqueous solution in vivo and to contribute to the response to the stimuli of reducing agents and low pH in the intracellular environment, leading to timely drug release. Besides, Zhang et al. employed terpyridine-metal coordination to prepare water-soluble monomers and suppressing cyclization, thus promoting supramolecular polymerization with both high molecular weight and good solubility [16]. The rigid structure and specific geometric fitting of CB[8] benefited the host-guest interaction between CB[8] and the naphthalene moieties of 1’’-(naphthalen-2-ylmethyl)-6’-(pyridin-2-yl)-[2,2’:4’,4’’-terpyridine]-1’’-ium bromide (NTPY), promoting supramolecular polymerization. Furthermore, the symmetrical and rigid structure of CBs leads to exceptional recognition properties, making these molecules as promising devices for supramolecular protein recognition. The high binding affinity of CBs to multiple residues on the protein surface results in changes in the protein conformation, showing their potential application in modulating the activity of biomolecules. Kim et al. reported mechanistic studies of the structural changes of ubiquitin (Ub) by host-guest chemistry with cucurbit[6]uril (CB[6]) [25]. The native structure of Ub contained an intraregional salt bridge between Lys33 and Thr14. As a result of the host-guest interaction between CB[6] and Lys11, the H-bond between Lys33 and Thr14 was broken, and a new H-bond was formed between Lys33 and Glu34.

Pillararene and calixarenes are cyclic oligomers formed by the condensation of phenols with formaldehyde under basic conditions, and they contain various numbers of rings. The upper and lower rims can be modified with multifunctional groups by conventional synthetic transformations, allowing a wide range of biological applications and leading to much significant progress in recent decades. Huang et al. made many remarkable contributions to improving the solution of macrocyclic compounds based on pillararenes and to design smart stimulus-responsive and low-toxicity structures for biotherapy. They reported a thermoresponsive supra-amphiphilic complexation based on Pillar[10]arene/Paraquat cooperative complexation [18]. The pillar[10]arene ring had two conformations in solution, which reacted to changes in the guest amount triggered by changes in temperature. These vesicles could be further used in the controlled release of small molecules, induced by cooling to 25 °C or heating to 60 °C. The same group also designed a new nanocontainer for in vivo tumor therapy by doping a water-soluble pillar[5]arene onto hollow mesoporous silica nanoparticles via host-guest complexation (shown in Figure 2B) [21]. Pillar[5]arene provided pH-sensitive storage and release of the drug and improved the biocompatibility while decreasing the toxicity, ultimately accelerating the inhibition of tumor growth in vivo with minimal side effects. As for calixarene, a series of supra-amphiphilic aggregates have been designed to directly assemble the small-molecule antipsychotic drug chlorpromazine (CPZ) into nanostructures with high loading efficiencies (shown in Figure 2C) [22,26]. CyDs consist of cyclic and interior cavity oligosaccharides and contain six to eight α-D-glucopyranoside units linked by α-1,4-glycosidic bonds. CyDs possess a hydrophobic cavity and can bind to various hydrophobically functionalized guest moieties, including docetaxel [27], gefitinib [28], resiquimod [29], MC11 peptide [30], pDNA [31], and miRNA-34a [32]. Arima et al. synthesized folic acid appended β-CyD possessing two caproic acids as a spacer and evaluated the potential of the resulting Fol-c2-β-CyD as a new tumor-targeting carrier [27]. The fluorescence intensity of doxorubicin (Dox) in the cells was clearly higher for Fol-c2-β-CyD than for Dox alone. After intratumoral administration to tumor-bearing mice, Dox/Fol-c2-β-CyD inhibited tumor growth relative to the control and to Dox alone because of the systemically favorable tumor targeting effect of the β-CyD derivatives and folic acid (FA). In addition, a synergistic enhancement therapy for lung cancer was developed with 2-hydroxypropyl-β-CyD modified with polyetherimide (PEI) (HP-β-CyD-PEI) [28]. The sequential delivery of pTSA and gefitinib (GFT) revealed a synergistic effect that can significantly enhance T cell immunity and promote cytokine production. Recently, much attention has been paid to modification of raw CyDs with responsive motifs, such as acetal and 4-phenylboronic acid pinacol ester, due to presence of many hydroxyl groups. Zhang et al. developed acetal modified CyDs with enhanced hydrophobicity, using emulsion method to construct spherical assemblies [33]. Acetal modified CyDs possessed acidic responsiveness and high biocompatibility with broad applications in areas such as nanomedicine and tissue engineering. Zhang et al. functionalized β-CyD with ROS liable 4-phenylboronic acid pinacol ester (PBAP), using emulsion method to formulate nanoparticles (NPs). The ROS-responsive nanoplatform could be hydrolyzed into raw β-CyD, attenuating the inflammation by PBAP and facilitating the subsequent drug release [34]. This β-CyD-based nanoplatform could be applied to treat chronic inflammation induced diseases, such as atherosclerosis [35] and abdominal aortic aneurysms [36].

In addition to the three most common kinds of cyclic oligomers, increasing attention has been paid to new rationally designed structures for biofunctions, especially the recognition of proteases and kinases. In studying metallo-supramolecular complexes, one enantiomer of chiral metallo-supramolecular complexes, NiP and NiM, has been synthesized as a telomerase inhibitor to eradicate cancer stem cells (CSCs) (shown in Figure 2D) [23]. Three ligands bridged a macrocyclic structure called NiP and NiM driven by the coordination between Ni atom and the nitrogen atoms of ligands. Traditional telomerase inhibitors killed cancer cells by telomere shortening, which required a long lag period and could activate the alternative lengthening of telomeres. In contrast, NiP could overcome these drawbacks by inducing and stabilizing the telomere G-quadruplex structure while its enantiomer, NiM, had little effect on cell growth in breast CSCs. Experimental results proved that the enantiomer NiP could effectively deplete breast CSCs in vitro and in vivo. Besides, potent macrocyclic pyrrolopyrimidines were also reported as MerTK inhibitors [37]. The macrocyclic pyrrolopyrimidines could form three hydrogen bonds with MerTK, two with the hinge region of the protein and the remaining one with R727. The X-ray structure of this macrocyclic compound complexed with MerTK was resolved and demonstrated that the macrocycles resided in the ATP binding pockets of the MerTK protein.

## 3. Biological Immunity Mechanisms in Vitro

**Figure 3:**
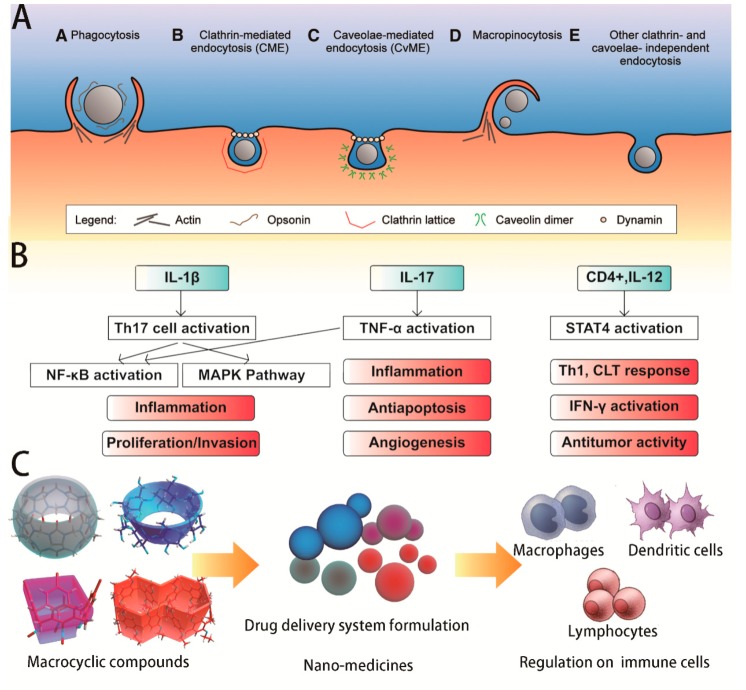
(**A**) Nanocarriers can be internalized into cells via several mechanisms, including phagocytosis and non-phagocytic pathways such as macropinocytosis, clathrin-mediated endocytosis (CME) and caveolae-mediated endocytosis (CvME). (Reprinted by permission from Springer Nature: Cellular and Molecular Life Sciences, Nanocarriers’ entry into the cell: relevance to drug delivery, Couvreur et al., 2009) [38]. (**B**) IL-1β enhances T cells activation and the development of Th17 cells, then activates the nuclear factor-κB (NF-κB) and MAPK-pathways, eventually leads to inflammation activation [39,40,41,42,43,44]; IL-17 activates the expression of tumor necrosis factor (TNF)-α in response to stimulation by lipopetides [45]; CD4^+^ T lymphocytes differentiate into Th1 cells induced by IL-12, then activating the expression of IFN-γ, finally inhibit tumor growth [46]. (**C**) Macrocyclic compounds based drug delivery systems for regulating function of immune cells (macrophages, dendritic cells, lymphocytes) [29,35,47,48,49].

Increasing attention has been paid to the mechanisms regulating the immune system. Indeed, most extraneous polymeric nanoparticles are rapidly cleared from the bloodstream within several minutes by macrophages of the reticuloendothelial system (RES) (shown in Figure 3A) [38]. Therefore many related contributions have focused on the control of particle physicochemical properties to overcome the limitations of drug and gene delivery in immune-modulating therapy.

### 3.1. Cell Uptake and Drug Delivery

To successfully gain access to intracellular pharmacological targets, therapeutic compounds must generally cross various biological membranes (e.g., the mucosa, epithelium, and endothelium) and diffuse through the plasma membrane. Nanocarriers can be internalized into cells via several mechanisms, including phagocytosis and non-phagocytic pathways such as macropinocytosis, clathrin-mediated endocytosis (CME) and caveolae-mediated endocytosis (CvME) [38].

As an indispensable physiological part of the defense against inert exogenous particles, phagocytosis occurs primarily in specialized cells: macrophages, monocytes, neutrophils and dendritic cells. The process can be described in three steps: first, recognition by opsonization in the bloodstream; second, adherence of the macrophages; and finally, ingestion by the host. Succinyl-β-CyD and L-lysine cross-linked CyD nanoparticles with a diameter of approximately 30 nm and a zeta potential of 0.90±1.90 mV were preferable for phagocytic uptake by macrophages [29]. Live attenuated Salmonellae coated with cationic polymers and plasmid DNA were captured by phagocytes within macrophages following invasion into the intestinal mucosa [31]. The resulting phagosome then matured into an acidified phagolysosome by the action of the vacuolar proton pump ATPase, leading to drug release.

Unlike phagocytosis, which is restricted to specialized cells, macropinocytosis is a type of clathrin-independent endocytosis pathway that requires no specific coating or concentration of receptors, so no selectivity is observed. In this mechanism, actin-driven membrane protrusions collapse onto and fuse with the plasma membrane to generate macropinosomes, which are generally larger than 1 μm and eventually fuse with a lysosome or recycle their content to the surface.

CME typically occurs in a membrane region enriched in clathrin, a coat protein with a three-legged structure called a triskelion. The macrocyclic compounds finally are degraded in an acidic and enzyme-rich environment. Li et al. developed a redox-sensitive gene vector targeting fibroblast growth factor receptors (FGFRs) [30]. The MC11 peptide (MQLPLATGGGC) could recognize FGFR and adhere to the surface of the cell membrane, where it was then ultimately ingested by CME. The complexes efficiently condensed pDNA into nanoparticles approximately 200 nm in diameter, an ideal design with an optimal phagocytosis rate and low adsorption of opsonins to escape the clearing by RES.

CvME has recently been identified as a highly regulated process, in contrast to CME. After fission from the membrane mediated by the GTPase dynamin, the vesicle can be delivered to a caveosome containing no enzymatic cocktail and thus can potentially avoid an acidic and enzyme-rich degradative environment. A rare-earth-incorporated polymeric vector was designed for enhanced gene delivery [50]. The decreased levels of calmodulin and a marked increase in the expression of caveolin indicated that the high transfection efficiency exhibited by the vector was attributed to the uptake proceeded via caveolae-mediated endocytosis.

### 3.2. Regulatory Cytokines and Signaling Pathways

Almost all immunological processes within proliferation and programmed cell death, as well as the maintenance of homeostasis of mammalian cells, are generally controlled by extracellular signals. These signals include a group of polypeptide growth and differentiation factors, lymphokines and monokines produced by activated T cells or macrophages, collectively called cytokines [51]. Cytokines are pleiotropic, and their significant functions make them natural targets for manipulation and clinical translation, particularly inflammasomes and proinflammatory cytokines, receptor tyrosine kinase and clusters of differentiation [52].

Inflammasomes are multi-protein complexes that include pattern recognition receptors able to recognize infectious agents and activate related signaling pathways (shown in Figure 3B). Latz et al. treated diet-induced atherosclerosis mice with CyD to investigate the efficacy of CyD treatment in murine atherosclerosis [47]. de Arrud et al. elucidated the anti-inflammatory effect of HP-2-β-CyD on monocytes isolated from HIV-positive and HIV-negative donors [53]. The plasma concentrations of IL-1β, which could enhance T cell activation and antigen recognition, induce the development of Th17 cells and ultimately activate the nuclear factor-κB (NF-κB) and mitogen-activated protein kinase (MAPK) pathways [39,40,41,42,43,44], were markedly reduced after treatment suggesting that CyD might reduce the inflammatory response.

Among proinflammatory cytokines, Toll-like receptors (TLRs), a class of proteins in the innate immune system, are individual, membrane-spanning and non-catalytic receptors usually expressed on macrophages and dendritic cells [54]. TNF-α primarily is primarily produced as a type II transmembrane arranged in stable homotrimers with the activation of NF-κB and p38-MAPK, which leads to cell death [55]. A new CB-containing nanostructure was developed to effectively deliver of IL-1Ra therapeutic protein, and resultantly inhibited IL-1β-induced pro-inflammatory cytokines production (such as TNF-α) [56].

Besides, small artificial macrocycles have been designed as potent IL17 antagonists different with the monoclonal antibodies currently on the market. Interleukin-17 (IL-17) is produced by activated memory T lymphocytes and stimulates innate immunity and host defense. In response to stimulation by lipopeptides, IL-17A localizes to a subset of T lymphocytes expressing tumor necrosis factor (TNF)-α [45]. Small artificial macrocycles exhibit numbers of advantages, such as low material costs and improved oral bioavailability and tissue penetration, leading to more effective treatments for IL-17A-related inflammatory diseases.

As for the receptor tyrosine kinases (RTKs), a 2-HP-β-CyD based cationic carrier was designed for the sequential delivery of gefitinib and a superantigen targeting epidermal growth factor receptor (EGFR) [28]. EGFR was one of four homologous transmembrane proteins that underwent nuclear translocalization and subsequently regulate gene expression [57]. The macrocyclic carrier revealed a synergistic effect of gefitinib and superantigen that significantly enhanced T-cell immunity, promoted TNF-α and IFN-γ production and inhibited tumor growth. In addition, a new redox-sensitive gene vector involving β-CyD was developed to target FGFRs [30]. FGFR, as a member of RTK family, could catalyze the transfer of the γ phosphate of ATP to the hydroxyl groups of tyrosines on target proteins, controlling most signaling pathways that involve fundamental cellular processes, including cell migration, metabolism and proliferation [58]. This vector showed better stabilization against extracellular salt aggregation, more effectively triggered release of DNA, and higher transfection efficiency than previous reported vectors.

Clusters of differentiation (CDs), grouped by their recognition of cell surface glycoproteins and the glycolipids of leukocyte molecules, have been widely used in research, diagnosis, and therapy [46]. In the major related studies, clusters of differentiation such as CD4^+^, CD8^+^and CD86 were evaluated to confirm the efficiency of drugs and vehicles. Furthermore, a new strategy was developed, for the first time, to efficiently deliver an oral DNA vaccine by live attenuated bacteria for efficacious cancer immunotherapy [31]. By coating the live attenuated bacteria with synthetic nanoparticles of CyDs and plasmid DNA, the nanoparticles could effectively escape phagosomes, encode autologous vascular endothelial growth factor receptor 2 (VEGFR2), and markedly activate the production of CD4^+^ and CD8^+^.

### 3.3. Regulation of Macrophage Reprogramming

Macrophages are the dominant members of the host immune system, and their rapid responses have attracted wide attention. 2-HP-β-CyD with an intrinsic property to solubilize cholesterol could promote liver X receptor (LXR)-mediated transcriptional reprogramming in macrophages [47]. Based on the excellent ability of macrophages to uptake excessive amounts of cholesterol, they examined how CyD influenced the ability of macrophages to process D6-cholesterol metabolism products. This analysis revealed that CyD treatment promoted the esterification of crystal-derived D6-cholesterol and increased the cholesterol efflux capacity of macrophages. More importantly, CyD was unexpectedly capable of increasing oxysterols production, which positively regulated the transcriptional activities of LXRs in macrophages, prompting a comprehensive investigation of the influence of CyD on the expression of LXR-regulated genes. The LXR target gene sets were enriched by CyD treatment in the wild type, while no enrichment was observed in LXRα^−/−^β^−/−^macrophages, confirming the CyD-induced expression of the target gene LXR in macrophages. 

### 3.4. Inducing the Maturation and Activation of Dendritic Cells and T Helper Lymphocytes

The induction of T helper (TH) lymphocytes by distinct TH ligands results in the generation of effector cells (shown in Figure 3C), namely, TH1 and TH2 cells, which are characterized by the expression of the cytokines interleukin (IL), interferon (IFN)-γ, and transforming growth factor (TGF)-β [59]. As for T follicular helper cells, occurring uniformly among B cells and a subset of CD4^+^ T cells, they play a crucial role in lymphocyte traffic within secondary lymphoid tissues and in the formation of the B-cell compartment [60]. The use of hydroxypropyl-β-CyD (HP-β-CyD) was reported to induce phenotypic and functional maturation of dendritic cells mainly mediated through lipid raft formation [48]. Furthermore, HP-β-CyD as a vaccine adjuvant could induce the response of Th2 cells and T follicular helper cells to the co-administered antigen [49]. To examine the activity of HP-β-CyD, C57BL/6 mice were s.c. administered LPS-free OVA with or without different doses of HP-β-CyD, and the OVA-specific total IgG, IgG1, and IgG2c titers were determined. All of the Ab titers were enhanced by all of the doses of HP-β-CyD tested. By further comparison with a typical Th2-type adjuvant (alum), the similar increases in Th2-type cytokines (IL-5 and IL-13) indicated that HP-β-CyD induced Th2 immune responses. To clarify its mode of action, a comprehensive mRNA Gene Chip analysis was performed, and the results showed that the expression of genes associated with the Th2-type immune response and the inflammatory response were upregulated in the draining LNs, spleen, and liver after injection. Tfh cells, a Th cell subset presenting in germinal centers (GCs) in LNs and influencing immune induction of immune, were investigated in LNs from Bcl6^f/+^ or Bcl6^f/f^ mice immunized with OVA or OVA/30% HP-β-CyD. After the second immunization, the generation of Tfh cells was significantly increased by HP-β-CyD in Bcl6^f/+^ mice and decreased in Bcl6^f/f^ mice, suggesting that Tfh cells were required for the adjuvant activity of HP-β-CyD. The activation of inflammasomes was also examined in vitro in macrophages and in vivo in Asc^−/−^ mice and Caspase 1^−/−^mice. However, no increase was observed in either gene-KO mice or in organs in vitro, clarifying that inflammasome activation was not essential for HP-β-CyD. Ultimately, further exploration found MyD88, a critical adapter protein of TLRs and the IL-1R superfamily, and TBK1, which regulated IFN-αβ receptors, to be involved in the adjuvant activity of HP-β-CyD.

## 4. Immune Therapy in Vivo

Based on abundant clarifications in vitro of the biological immunity mechanisms of macrocyclic compounds, further investigations in vivo have focus on immunotherapy for different diseases to develop potential applications of macrocyclic compounds in clinical therapy (Table 1).

### 4.1. Cancer Treatment

**Figure 4:**
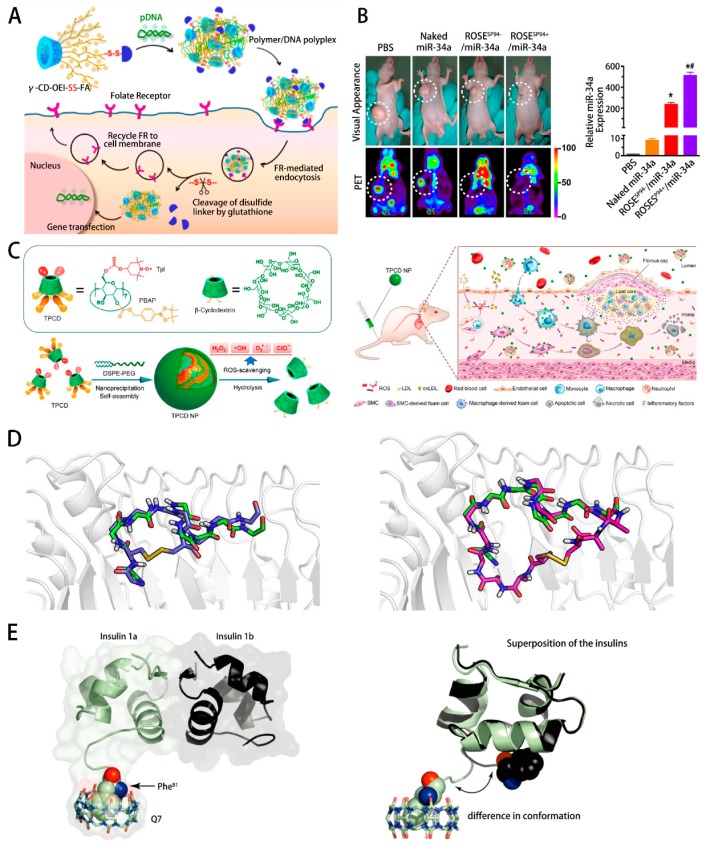
(**A**) The preparation of folic acid modified nanoparticles and schematic illustration of drug-release process reported by Li et al. (Reprint with permission from Biomacromolecules 2013, 14, 476–484. Copyright © 2013, American Chemical Society) [69]. (**B**) The anti-tumor therapy of redox-sensitive, oligopeptide-guided, self-assembling, and efficiency-enhanced (ROSE) system reported by Tang et al. (Reprinted from Biomaterials, 104, Tang et al., A redox-sensitive, oligopeptide-guided, self-assembling, and efficiency-enhanced (ROSE) system for functional delivery of microRNA therapeutics for treatment of hepatocellular carcinoma, 192–200, 2016, with permission from Elsevier) [32]. (**C**) Targeted therapy of atherosclerosis by abroad-spectrum reactive oxygen species scavenging nanoparticle with intrinsic anti-inflammatory activity reported by Zhang et al. (Reprint with permission from ACS Nano 2018, 12, 8943–8960. Copyright © 2018, American Chemical Society) [35]. (**D**) Two rationally designed macrocyclic peptides as synergistic agonists of LPS-induced inflammatory response reported by Yin et al. (Reprinted from Tetrahedron, 70, Yin et al., Rationally designed macrocyclic peptides as synergistic agonists of LPS-induced inflammatory response, 7664–7668, 2014, with permission from Elsevier) [70]. (**E**) Crystal structure of cucurbit[7]uril with insulin reported by Urbach et al. (Reprint with permission from J. Am. Chem. Soc. 2011, 133, 8810–8813. Copyright © 2011, American Chemical Society) [71].

Increasingly extensive studies on macrocyclic chemistry have been conducted to contribute to the understanding and treatment of cancer. For example, Li et al. synthesized a smart star-shaped cationic polymer containing a γ-CyD core and multiple oligoethylenimine (OEI) arms with folic acid (FA) linked by a bio-reducible disulfide bond for efficient targeted gene delivery (shown in Figure 4A) [69]. The newly cationic polymer could be cleaved efficiently and readily released FA under reductive intracellular conditions. A high density of folate receptor (FR) on the cellular membrane facilitated continuous FR-mediated endocytosis, leading to very high levels of gene expression. The gene transfection efficiency of γ-CyD-OEI-SS-FA_1.3_ was found to be up to 2-fold higher than that of γ-CyD-OEI-SS-FA_1.2_, demonstrating that the amount of FA had a significant influence on the smart FR-recycling function. Additionally, Tang et al. designed a novel redox-sensitive, oligopeptide-guided, self-assembling, efficiency-enhanced (ROSE) system to deliver the tumor suppressor miR-34a for hepatocellular carcinoma (HCC) (shown in Figure 4B) [32]. Conclusively, the ROSE system showed the most effective intratumoral accumulation, the highest miR-34a expression (approximately 500-fold greater than that of the PBS treatment setting), and significant inhibition of tumor growth, resulting in the lowest tumor size of 41.16 mm^3^ among all formulations. Besides, a proof-of-concept work in oral DNA vaccines is worth recalling [31]. Lived attenuated Salmonellae were coated with nanoparticles self-assembled from cationic polymers and plasmid DNA for the first time, greatly promoting infection efficiency and immunotherapy with T-cell activation and cytokine production. The protective nanoparticle coating layer is able to facilitate bacteria to effectively escape phagosomes, significantly enhance the acid tolerance of bacteria in stomach and intestines, and greatly promote dissemination of bacteria into blood circulation after oral administration. 

### 4.2. Treatment of Cardiovascular and Other Diseases

Atherosclerosis is an inflammatory disease linked to elevated blood cholesterol concentrations, which may lead to the occurrence of some cardiovascular disease including coronary artery disease, stroke, and peripheral artery. 2-HP-β-CyD was used to explore its antiatherogenic effects in vivo (shown in Figure 3B) [47]. In the diet-induced atherosclerosis model of apolipoprotein E-deficient (ApoE^−/−^) mice, the amounts of cholesterol crystals (CCs) in atherosclerotic plaques were assessed by laser reflection microscopy. A clear reduction in CCs was observed in the atherosclerotic plaques, verifying that preliminary 2-HP-β-CyD treatment could impair atherogenesis in vivo. Then they tested 2-HP-β-CyD-induced macrophage cholesterol efflux in vivo via bone marrow transplantation studies. Notably, 2-HP-β-CyD treatment resulted in a marked increase in D6-cholesterol excretion into the feces and in urinary elimination. To investigate the stimulation of urinary cholesterol excretion by 2-HP-β-CyD in humans, three individual patients with NPC1 mutations were monitored upon the intravenous application of 2-HP-β-CyD, which resulted in time-dependent cholesterol excretion into the urine. All these data suggested that 2-HP-β-CyD enhances reverse cholesterol transport (RCT) in vivo. Recently, α-CyD was also used to explore its potential role in managing atherosclerosis in vitro [72]. α-CyD strongly inhibited CC-induced complement activation by inhibiting binding of the pattern recognition molecules C1q (via IgM) and fcolin-2. The reduced CC-induced complement activation mediated by α-CyD resulted in reduced phagocytosis and reduced ROS production in monocytes and granulocytes. α-CyD was the most effective inhibitor of CC-induced complement activation, with the reduction in deposition of complement activation products being significantly different from the reduction induced by 2-hydroxypropyl-β-CyD. They also found that α-CyD could dissolve CCs in vitro. All results showed that superior complement inhibitory effects of α-CyD on CC-induced inflammation compared to 2-HP-β-CyD.

For Niemann-Pick type C1 disease (NPC1), CyD was reported to overcome the transport defect in nearly every organ of NPC1 mice, leading to the excretion of sequestered cholesterol as bile acid [73]. The acute administration of 2-hydroxypropyl-β-CyD could reverse the intracellular transport defect in both young and mature NPC1^−/−^mice. And these effects were observed in the liver, brain and peripheral organs including the spleen and kidney, but not the lung. Furthermore, various applications to other diseases were possible. For example, the co-administration of rationally designed macrocyclic peptides with lipopolysaccharide (LPS) could regulate Toll-like receptor (TLR)-mediated inflammatory response (shown in Figure 4D) [70]. The macrocyclic peptides contained disulfide-bond forming cysteine residues, which were similar to the myeloid differentiation factor 2 (MD2)-binding region of TLR. They worked synergistically with LPS in induction of TLR4 signaling and influenced the production of a number of proinflammatory cytokines. And the synthetic receptor cucurbit[7]uril (CB[7]) could specifically bind to insulin (shown in Figure 4E), offering potential for the treatment of diabetes [71], among other possibilities [74,75,76,77]. The nonpolar interior and carbonyl-lined portals of CB[7] receptors worked in concert to bind short peptides with an N-terminal phenylalanine (Phe). Human insulin exactly contained a rare Phe residue at the N-terminus of its B-chain so that CB[7]could specifically bind to human insulin efficiently.

## 5. Conclusions and Outlook

Various research accomplishments involving macrocyclic compounds for drug and gene delivery in immune-modulating therapies have been summarized. As native macrocyclic compound with high biocompatibility, CyDs have been approved by the Food and Drug Administration (FDA) and are widely used as drug excipient. And the multiple hydroxyl functional groups of CyDs, CyDs-based nanomedicines have been constructed and intrinsic biological functions have been discovered, which may stimulate deeper study of CyDs based nanomedicines. Cucurbiturils (CBs), calixarenes, pillararenes and other new structures such as macrocyclic peptides and metallo-supramolecular compounds have been introduced briefly in terms of their physicochemical properties and representative constructions. Numerous studies in vitro and in vivo have been performed regarding biological immunity mechanisms from the cell internalization to dominant cytokines in both innate immunity and adaptive immunity. Although the metabolomics of macrocyclic compounds remain unclear, we believe that a bright future lies ahead for the use of macrocyclic compounds in immune therapy.

## Figures and Tables

**Table 1:** Macrocyclic compound-based therapeuticsystems.

Macrocyclic Compound	Drug	Use	Method	Outcome	Ref.
β-CyD	MC11 peptide	SKOV-3 cancer	Physical mixture	Targeted FGFRs and improved transfection efficiency	[30]
β-CyD	pDNA	B16 melanoma tumor	Physical mixture	Promoted infection efficiency and immunotherapy, including T cell activation and cytokine production	[31]
β-CyD	Meloxicam	Infectious arthritis	Physical mixture or kneading	Enhanced the solubility and stability of meloxicam for anti-inflammatory and analgesic effects	[61]
β-CyD	Etodolac	Infectious arthritis	Physical mixture	Anti-inflammatory	[62]
2-hydroxypropyl-β-CyD	MicroRNA-34a	HCC-LM3 tumor	Lyophilization	Redox-sensitive, oligopeptide-guided, self-assembling, efficiency-enhanced system for the treatment of hepatocellular carcinoma	[32]
2-hydroxypropyl-β-CyD		Atherosclerosis		Increased oxysterol production and promoted macrophage reprogramming	[47]
2-hydroxypropyl-β-CyD	Budesonide	Ulcerative colitis	Lyophilization	Improved the solubility of budesonide and benefited the treatment of ulcerative colitis	[63]
sulfobutyl ether-β-CyD	Resveratrol	MCF-7 tumor	Freeze-drying	Increased the solubility and anticancer activity of resveratrol	[64]
cucurbit[8]uril	Doxorubicin	HeLa tumor	Lyophilization	Self-imaging and controllable drug release ability	[20]
cucurbit[7]uril	Zoledronic and Doxorubicin	MCF-7 tumor	Physical mixture	Sequential delivery of doxorubicin/zoledronic acid to breast cancer cells	[65]
pillar[6]arene	Tamoxifen	MCF-7 and Hela tumor	Physical mixture	Enhanced the solubility and bioactivity of the poorly water-soluble anticancer drug tamoxifen	[66]
pillar[6]arene	Chlorambucil	A549 tumor	Physical mixture	Photocontrolled anticancer drug release	[67]
calix[8]arenes with glycosylation		MCF-7 tumor	Physical mixture	Prevented tumor migration and proliferation	[68]

## References

[1] Chen H.B., Gu Z.J., An H.W., Chen C.Y., Chen J., Cui R., Chen S.Q., Chen W.H., Chen X.S., Chen X.Y. (2018). Precise nanomedicine for intelligent therapy of cancer. Sci. China Chem..

[2] Yang G.B., Phua S.Z.F., Bindra A.K., Zhao Y.L. (2019). Degradability and Clearance of Inorganic Nanoparticles for Biomedical Applications. Adv. Mater..

[3] Fang R.H., Kroll A.V., Gao W.W., Zhang L.F. (2018). Cell Membrane Coating Nanotechnology. Adv. Mater..

[4] Maruf A., Wang Y., Yin T.Y., Huang J.L., Wang N., Durkan C., Tan Y.H., Wu W., Wang G.X. (2019). Atherosclerosis Treatment with Stimuli-Responsive Nanoagents: Recent Advances and Future Perspectives. Adv. Healthc. Mater..

[5] Nguyen T.L., Choi Y., Kim J. (2018). Mesoporous Silica as a Versatile Platform for Cancer Immunotherapy. Adv. Mater..

[6] Mishra P.K., Mishra H., Ekielski A., Talegaonkar S., Vaidya B. (2017). Zinc oxide nanoparticles: A promising nanomaterial for biomedical applications. Drug Discov. Today.

[7] Mishra H., Mishra P.K., Ekielski A., Jaggi M., Iqbal Z., Talegaonkar S. (2018). Melanoma treatment: From conventional to nanotechnology. J. Cancer Res. Clin. Oncol..

[8] Bhandari J., Mishra H., Mishra P.K., Wimmer R., Ahmad F.J., Talegaonkar S. (2017). Cellulose nanofiber aerogel as a promising biomaterial for customized oral drug delivery. Int. J. Nanomed..

[9] Zhou J., Yu G.C., Huang F.H. (2017). Supramolecular chemotherapy based on host–guest molecular recognition: A novel strategy in the battle against cancer with a bright future. Chem. Soc. Rev..

[10] Harada A., Takashima Y., Yamaguchi H. (2009). Cyclodextrin-based supramolecular polymers. Chem. Soc. Rev..

[11] Harada A. (2001). Cyclodextrin-Based Molecular Machines. Acc. Chem. Res..

[12] Harada A., Takashima Y., Nakahata M. (2014). Supramolecular Polymeric Materials via Cyclodextrin−Guest Interactions. Acc. Chem. Res..

[13] Bogdan A.R., Davies N.L., James K. (2011). Comparison of diffusion coefficients for matched pairs of macrocyclic and linear molecules over a drug-like molecular weight range. Org. Biomol. Chem..

[14] Mallinson J., Collins I. (2012). Macrocycles in new drug discovery. Future Med. Chem..

[15] Driggers E.M., Hale S.P., Lee J.B., Terrett N.K. (2008). The exploration of macrocycles for drug discovery-an underexploited structural class. Nat. Rev. Drug Discov..

[16] Liu Y.L., Huang Z.H., Tan X.X., Wang Z.Q., Zhang. X. (2013). Cucurbit[8]uril-based supramolecular polymers: Promoting supramolecular polymerization by metal-coordination. Chem. Commun..

[17] Gallego-Yerga L., Lomazzi M., Sansone F., Mellet C.O., Casnatib A., Ferna´ndez J.M.G. (2014). Glycoligand-targeted core-shell nanosphereswith tunable drug release profiles from calixarene-cyclodextrin heterodimers. Chem. Commun..

[18] Chi X.D., Yu G.C., Shao L., Chen J.Z., Huang F.H. (2016). A Dual-Thermoresponsive Gemini-Type Supra-amphiphilic Macromolecular [3] Pseudorotaxane Based on Pillar[10]arene/Paraquat Cooperative Complexation. J. Am. Chem. Soc..

[19] Ping Y., Liu C.D., Zhang Z.X., Liu L.K., Chen J.H., Li J. (2011). Chitosan-graft-(PEI-β-cyclodextrin) copolymers and their supramolecular PEGylation for DNA and siRNA delivery. Biomaterials.

[20] Wu D., Li Y., Yang J., Shen J., Zhou J., Hu Q.L., Yu G.C., Tang G.P., Chen X.Y. (2017). Supramolecular Nanomedicine Constructed from Cucurbit[8]uril-Based Amphiphilic Brush Copolymer for Cancer Therapy. ACS Appl. Mater. Interfaces.

[21] Yao Y., Wang Y., Zhao R.B., Shao L., Tang R.K., Huang F.H. (2016). Improved in vivo tumor therapy via host–guest complexation. J. Mater. Chem. B..

[22] Qin Z.B., Guo D.S., Gao X.N., Liu Y. (2014). Supra-amphiphilic aggregates formed by psulfonatocalix[4]arenes and the antipsychotic drug chlorpromazine. Soft Matter..

[23] Qin H.S., Zhao C.Q., Sun Y.H., Ren J.S., Qu X.G. (2017). Metallo-supramolecular complexes enantioselectively eradicate cancer stem cells in vivo. J. Am. Chem. Soc..

[24] Masson E., Ling X.X., Joseph R., Kyeremeh-Mensah L., Lu X.Y. (2012). Cucurbituril chemistry: A tale of supramolecular success. RSC Adv..

[25] Lee J.W., Heo S.W., Lee S.J.C., Ko J.Y., Kim H., Kim H.I. (2013). Probing Conformational Changes of Ubiquitin by Host–Guest Chemistry Using Electrospray Ionization Mass Spectrometry. J. Am. Soc. Mass Spectrom.

[26] Wang Y.X., Guo D.S., Cao Y., Liu Y. (2013). Phosphatase-responsive amphiphilic calixarene assembly. RSC Adv..

[27] Okamatsu A., Motoyama K., Onodera R., Higashi T., Koshigoe T., Shimada Y., Hattori K., Takeuchi T., Arima H. (2013). Folate-Appended β-Cyclodextrin as a Promising Tumor Targeting Carrier for Antitumor Drugs in Vitro and in Vivo. Bioconjugate Chem..

[28] Li D., Li Y.B., Xing H.B., Guo J.L., Yuan P., Tang G.P. (2014). Synergistic Enhancement of Lung Cancer Therapy Through Nanocarrier-Mediated Sequential Delivery of Superantigen and Tyrosin Kinase Inhibitor. Adv. Funct. Mater..

[29] Rodell C.B., Arlauckas S.P., Cuccarese M.F., Garris C.S., Li R., Ahmed M.S., Kohler R.H., Pittet M.J., Weissleder R. (2018). TLR7/8-agonist-loaded nanoparticles promote the polarization of tumour-associated macrophages to enhance cancer immunotherapy. Nat. Biomed. Eng..

[30] Ping Y., Hu Q.D., Tang G.P., Li J. (2013). FGFR-targeted gene delivery mediated by supramolecular assembly between β-cyclodextrin-crosslinked PEI and redox-sensitive PEG. Biomaterials.

[31] Hu Q.L., Wu M., Fang C., Cheng C.Y., Zhao M.M., Fang W.H., Chu P.K., Ping Y., Tang G.P. (2015). Engineering Nanoparticle-Coated Bacteria as Oral DNA Vaccines for Cancer Immunotherapy. Nano Lett..

[32] Hu Q.D., Wang K., Sun X., Li Y., Fu Q.H., Liang T.B., Tang G.P. (2016). A redox-sensitive, oligopeptide-guided, self-assembling, and efficiency-enhanced (ROSE) system for functional delivery of microRNA therapeutics for treatment of hepatocellular carcinoma. Biomaterials.

[33] Zhang J.X., Jia Y., Li X.D., Hu Y.Q., Li X.H. (2011). Facile Engineering of Biocompatible Materials with pH-Modulated Degradability. Adv. Mater..

[34] Zhang D.L., Wei Y.L., Chen K., Zhang X.J., Xu X.Q., Shi Q., Han S.L., Chen X., Gong H., Li X.H. (2015). Biocompatible Reactive Oxygen Species (ROS) Responsive Nanoparticles as Superior Drug Delivery Vehicles. Adv. Healthcare Mater..

[35] Wang Y.Q., Li L.L., Zhao W.B., Dou Y., An H.J., Tao H., Xu X.Q., Jia Y., Lu S., Zhang J.X. (2018). Targeted Therapy of Atherosclerosis by a Broad-Spectrum Reactive Oxygen Species Scavenging Nanoparticle with Intrinsic Anti-inflammatory Activity. ACS Nano.

[36] Cheng J., Zhang R.J., Li C.W., Tao H., Dou Y., Wang Y.Q., Hu H.Y., Zhang J.X. (2018). A Targeting Nanotherapy for Abdominal Aortic Aneurysms. J. Am. Coll. Cardiol..

[37] Wang X.D., Liu J., Zhang W.H., Stashko M.A., Nichols J., Miley M.J., Norris-Drouin J., Chen Z.L., Machius M., DeRyckere D. (2016). Design and Synthesis of Novel Macrocyclic Mer Tyrosine Kinase Inhibitors. ACS Med. Chem. Lett..

[38] Hillaireau H., Couvreur P. (2009). Nanocarriers’ entry into the cell: Relevance to drug delivery. Cell. Mol. Life Sci..

[39] Sahoo M., Ceballos-Olvera I., Barrio L.D., Re F. (2011). Role of the Inflammasome, IL-1β, and IL-18 in Bacterial Infections. Sci. World J..

[40] Netea M.G., van de Veerdonk F.L., van der Meer J.M., Dinarello C., Joosten L.B. (2015). Inflammasome-Independent Regulation of IL-1-Family Cytokines. Annu. Rev. Immunol..

[41] Hayden M.S., Ghosh S. (2004). Signaling to NF-κB. Genes Dev..

[42] Li Q.T., Verma I.M. (2002). NF-κB Regulation in the immune system. Nat. Rev. Immunol..

[43] Chang L.F., Karin M. (2001). Mammalian MAP kinase signalling cascades. Nature.

[44] Roux P.P., Blenis J. (2004). ERK and p38 MAPK-Activated Protein Kinases: A Family of Protein Kinases with Diverse Biological Functions. Microbiol. Mol. Biol. Rev..

[45] Kolls J.K., Linde A. (2004). Interleukin-17 Family Members and Inflammation. Immunity.

[46] Zola H., Swart B., Nicholson I., Aasted B., Bensussan A., Boumsell L., Buckley C., Clark G., Drbal K., Engel P. (2005). CD molecules 2005: Human cell differentiation molecules. Blood.

[47] Zimmer S., Grebe A., Bakke S.S., Bode N., Halvorsen B., Ulas T., Skjelland M., Nardo D.D., Labzin L.I., Kerksiek A. (2016). Cyclodextrin promotes atherosclerosis regression via macrophage reprogramming. Sci. Transl. Med..

[48] Kim S.K., Yun C.H., Han S.H. (2016). Induction of Dendritic cell Maturation and activation by a Potential adjuvant, 2-hydroxypropyl-β-cyclodextrin. Front. Immunol..

[49] Onishi M., Ozasa K., Kobiyama K., Ohata K., Kitano M., Taniguchi K., Homma T., Kobayashi M., Sato A., Katakai Y. (2015). Hydroxypropyl-β-Cyclodextrin Spikes Local Inflammation That Induces Th2 Cell and T Follicular Helper Cell Responses to the Coadministered Antigen. J. Immunol..

[50] Wang Q.W., Jin W.H., Wu G.S., Zhao Y., Jin X., Hu X.R., Zhou J., Tang G.P., Chu P.K. (2014). Rare-earth-incorporated polymeric vector for enhanced gene delivery. Biomaterials.

[51] Arai K., Lee F., Miyajima A., Miyatake S., Arai N., Yokota T. (1990). Cytokines: Coordinators of immune and inflammatory responses. Annu. Rev. Biochem..

[52] Stenken1 J.A., Poschenrieder A.J. (2015). Bioanalytical Chemistry of Cytokines-A Review. Anal. Chim. Acta.

[53] Matassoli F.L., Leão I.C., Bezerra B.B., Pollard R.B., Lütjohann D., Hildreth J.E.K., de Arruda L.B. (2018). Hydroxypropyl-Beta-Cyclodextrin Reduces Inflammatory Signaling from Monocytes: Possible Implications for Suppression of HIV Chronic Immune Activation. mSphere.

[54] Takeda K., Akira S. (2005). Toll-like receptors in innate immunity. Int. Immunol..

[55] Wajant H., Pfizenmaier K., Scheurich P. (2003). Tumor necrosis factor signaling. Cell Death Differ..

[56] Wang T.T., Tang Y.Q., He X., Yan J., Wang C.H., Feng X.L. (2017). Self-Assembled Raspberry-Like Core/Satellite Nanoparticles for Anti-Inflammatory Protein Delivery. ACS Appl. Mater. Interfaces.

[57] Lo H.W., Hung M.C. (2006). Nuclear EGFR signalling network in cancers: Linking EGFR pathway to cell cycle progression, nitric oxide pathway and patient survival. Br. J. Cancer.

[58] Schlessinger J. (2000). Cell Signaling by Receptor Tyrosine Kinases. Cell.

[59] Yu Q.L., Watson R.R., Marchalonis J.J., Larson D.F. (2005). A role for T lymphocytes in mediating cardiac diastolic function. Am J Physiol Heart Circ Physiol..

[60] Moser B., Schaerli P., Loetscher P. (2002). CXCR5+ T cells: Follicular homing takes center stage in T-helper-cell responses. Trends Immunol..

[61] Shende P.K., Gaud R.S., Bakal R., Patil D. (2015). Effect of inclusion complexation of meloxicam with β-cyclodextrin and β-cyclodextrin-based nanosponges on solubility, in vitro release and stability studies. Colloids Surf. B.

[62] Puglisi A., Rizzarelli E., Vecchio G., Iacovino R., Benedetti E., Pedone C., Saviano M. (2009). Crystal and Molecular Structure of β-Cyclodextrins Functionalized with the Anti-Inflammatory Drug Etodolac. Biopolymers.

[63] Akkari A.C.S., Campos E.V.R., Keppler A.F., Fraceto L.F., de Paula E., Tófoli G.R., de Araujo D.R. (2016). Budesonide-hydroxypropyl-β-cyclodextrin inclusion complex in binary poloxamer 407/403 system for ulcerative colitis treatment: A physico-chemical study from micelles to hydrogels. Colloids Surf. B.

[64] Venuti V., Cannavà C., Cristiano M.C., Fresta M., Majolino D., Paolino D., Stancanelli R., Tommasini S., Ventura C.A. (2014). A characterization study of resveratrol/sulfobutyl ether-β-cyclodextrin inclusion complex and in vitro anticancer activity. Colloids Surf. B.

[65] Benyettou F., Alhashimi M., O’Connor M., Pasricha R., Brandel J., Traboulsi H., Mazher J., Olsen J.C., Trabolsi A. (2017). Sequential Delivery of Doxorubicin and Zoledronic Acid to Breast Cancer Cells by CB[7]-Modified Iron Oxide Nanoparticles. ACS Appl. Mater. Interfaces.

[66] Shangguan L.Q., Chen Q., Shi B.B., Huang F.H. (2017). Enhancing the solubility and bioactivity of anticancer drug tamoxifen by water-soluble pillar[6]arene-based host–guest complexation. Chem. Commun..

[67] Yu G.C., Yu W., Mao Z.W., Gao C.Y., Huang F.H. (2015). A Pillararene-Based Ternary Drug-Delivery System with Photo controlled Anticancer Drug Release. Small.

[68] Geraci C., Consoli G.M.L., Granata G., Galante E., Palmigiano A., Pappalardo M., Di Puma S.D., Spadaro A. (2013). First Self-Adjuvant Multicomponent Potential Vaccine Candidates byTethering of Four or Eight MUC1 Antigenic Immunodominant PDTRPUnits on a Calixarene Platform: Synthesis and Biological Evaluation. Bioconjugate Chem..

[69] Zhao F., Yin H., Zhang Z.X., Li J. (2013). Folic Acid Modified Cationic γ-Cyclodextrin-oligoethylenimine Star Polymer with Bioreducible Disulfide Linker for Efficient Targeted Gene Delivery. Biomacromolecules.

[70] Gao M., London N., Cheng K., Tamura R., Jin J.L., Schueler-Furman O., Yin H. (2014). Rationally designed macrocyclic peptides as synergistic agonists of LPS-induced inflammatory response. Tetrahedron.

[71] Chinai J.M., Taylor A.B., Ryno L.M., Hargreaves N.D., Morris C.A., Hart P.J., Urbach A.R. (2011). Molecular Recognition of Insulin by a Synthetic Receptor. J. Am. Chem. Soc..

[72] Pilely K., Bakke S.S., Palarasah Y., Skjoedt M.O., Bartels E.D., Espevik T., Garred P. (2019). Alpha-cyclodextrin inhibits cholesterol crystal-induced complement mediated inflammation: A potential new compound for treatment of atherosclerosis. Atherosclerosis.

[73] Liu B., Ramirez C.M., Miller A.M., Repa J.J., Turley S.D., Dietschy J.M. (2010). Cyclodextrin overcomes the transport defect in nearly every organ of NPC1 mice leading to excretion of sequestered cholesterol as bile acid. J. Lipid Res..

[74] Zadmard R., Alavijeh N.S. (2014). Protein surface recognition by calixarenes. RSC Adv..

[75] Giuliani M., Morbioli I., Sansone F., Casnati A. (2015). Moulding calixarenes for biomacromolecule targeting. Chem. Commun..

[76] Gordo S., Martos V., Santos E., Mene´ndez M., Bo C., Giralt E., de Mendoza J. (2008). Stability and structural recovery of the tetramerization domain of p53-R337H mutant induced by a designed templating ligand. Proc. Natl. Acad. Sci. USA.

[77] Xu Z., Jia S.R., Wang W., Yuan Z., Ravoo B.J., Guo D.S. (2019). Heteromultivalent peptide recognition by co-assembly of cyclodextrin and calixarene amphiphiles enables inhibition of amyloid fibrillation. Nat. Chem..

